# Supplementary material for: The Prognostic Value of Pre-Treatment Circulating Biomarkers of Systemic Inflammation (CRP, dNLR, YKL-40, and IL-6) in Vulnerable Older Patients with Metastatic Colorectal Cancer Receiving Palliative Chemotherapy—The Randomized NORDIC9-Study
Source: J Clin Med. 2022 Sep 23;11(19):5603. doi: 10.3390/jcm11195603 (PMC9571053; doi:10.3390/jcm11195603)
Supplement: Supplementary file 1 [file jcm-11-05603-s001.zip › jcm-1891858-supplementary.pdf]

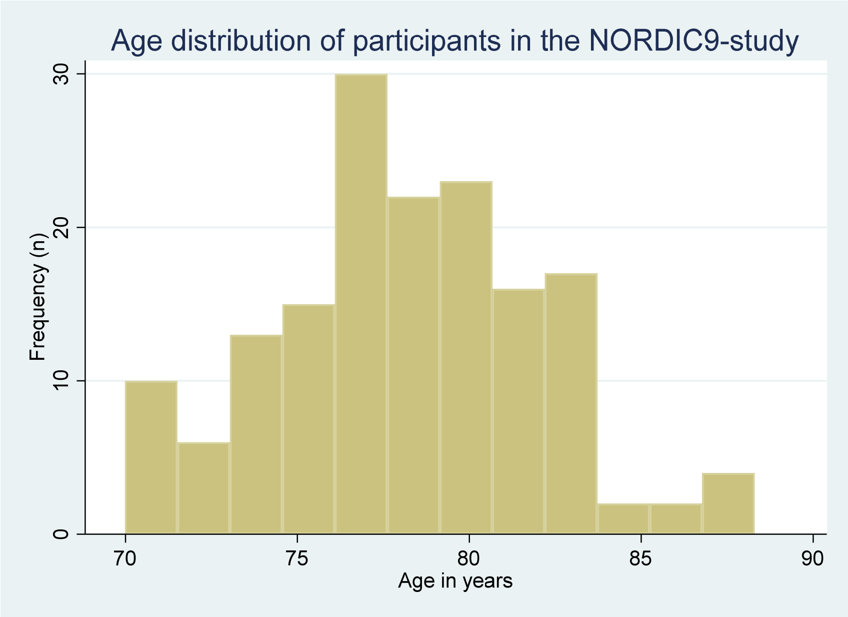

Supplementary Figure S1. Age distribution of older fragile patients with metastatic colorectal cancer included in the randomized NORDIC9-study.

Biomarker	n	Progression-free Survival			Overall Survival		
		Hazard ratio	95% CI	p-value	Hazard ratio	95% CI	p-value
**CRP** (mg/L)							
≤ 10	66	1.85	(1.32-2.58)	**<0.001**	3.36	(2.23-5.08)	**<0.001**
> 10	86						
**dNLR**							
≤ 2.2	79	1.42	(1.03-1.96)	**0.034**	1.89	(1.29-2.75)	**0.001**
> 2.2	78						
**YKL-40** (µg/L)							
≤ 200	72	1.40	(0.97-2.00)	0.069	1.81	(1.20-2.74)	**0.005**
> 200	56						
**IL-6** (ng/L)							
≤ 4.5	47	1.52	(1.04-2.21)	**0.030**	1.55	(0.99-2.42)	0.053
> 4.5	81						

Supplementary Table S1. Univariate cox analysis of inflammatory biomarkers CRP, dNLR, YKL-40, and IL-6 on progression-free survival and overall survival in the NORDIC9-study.

NA: Non-applicable.

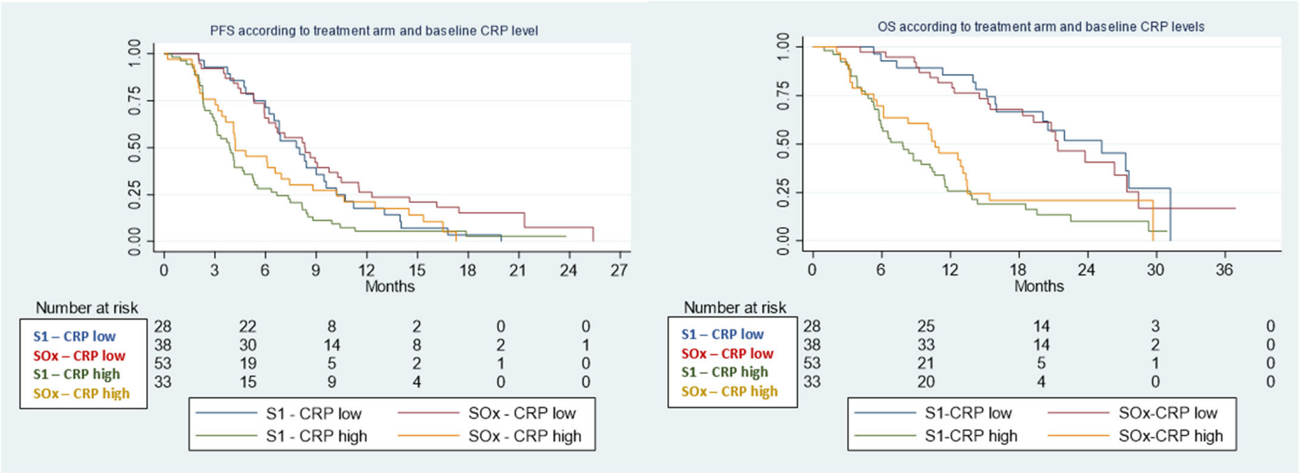

Supplementary Figure S2. Kaplan-Meier survival curves demonstrating progression-free survival and overall survival stratified by treatment arm and baseline CRP levels.

**Progression-free survival**				**Overall Survival**			
**Treatment arm - CRP level**	**Median PFS (months) 95% CI**	**Hazard ratio 95% CI**	**p-value**	**Treatment arm - CRP level**	**Median OS (months) 95% CI**	**Hazard ratio 95% CI**	**p-value**
S1- CRP low	7.8<br>6.2-9.5	1.00	NA	S1- CRP low	25.2<br>15.9-NC	1.00	NA
SOx – CRP low	8.3<br>5.9-10.3	0.74<br>0.44-1.23	0.242	SOx – CRP low	21.4<br>18.3-27.4	1.09<br>0.57-2.08	0.805
S1 – CRP high	**3.9**<br>**2.9-4.7**	**1.82**<br>**1.14-2.89**	**0.012**	S1 – CRP high	**7.8**<br>**5.8-10.4**	**3.85**<br>**2.15-6.90**	**<0.001**
SOx – CRP high	4.2<br>3.4-6.9	1.26<br>0.75-2.10	0.379	SOx – CRP high	**10.6**<br>**6.1-13.3**	**3.07**<br>**1.62-5.79**	**0.001**

Supplementary Table S2. Progression-free survival and overall survival according to treatment arm and baseline CRP levels. Overall survival was significant shorter in patients with high CRP levels regardless treatment arm.
NA: not applicable
NC: not calculated
